# Potential Impact of *Sclerocarya birrea* on Cardiovascular Health and Related Risk Factors: Review of Existing Evidence

**DOI:** 10.3390/antiox14080997

**Published:** 2025-08-14

**Authors:** Given R. Mashaba, Kabelo Mokgalaboni, Sogolo L. Lebelo

**Affiliations:** 1 DIMAMO Population Health Research Centre, University of Limpopo, Sovenga 0727, South Africa; 2 Department of Life and Consumer Sciences, College of Agriculture and Environmental Sciences, University of South Africa, Roodepoort 1710, South Africa; mokgak@unisa.ac.za

**Keywords:** *Sclerocarya birrea*, antidiabetic, hypolipidemic, antihypertensive, antioxidative, cardiovascular health

## Abstract

There is increasing use of modern medicine globally to manage cardiovascular diseases (CVDs). However, many people, especially in low-to-middle-income countries, still rely on traditional medicinal plants for their daily health needs. However, limited studies have explored the use of these remedies. Therefore, this narrative review aimed to evaluate the potential of *Sclerocarya birrea* (*S. birrea*) in managing diabetes, dyslipidemia, inflammation, and hypertension, including its effects on oxidative stress. This study reviewed evidence from PubMed, Web of Science, and ResearchGate, published in these databases up to 30 April 2025. The evidence showed that *S. birrea* had the potential to preserve cardiometabolic health and reduce CVD-associated risk factors. Notably, *S. birrea* improved glucose metabolism, inflammation, hypertension, and oxidative stress. This plant exhibits antihyperglycemic effects by activating adenosine monophosphate-activated protein kinase (AMPK) and inhibiting gluconeogenesis and the activities of carbohydrase. It also ameliorates dyslipidemia by modulating the activities of peroxisome proliferator-activated receptor alpha (PPARα) and increasing fatty acid oxidation. The anti-inflammatory potential of *S. birrea* is modulated by the activation of PPARα, which inhibits nuclear factor kappa beta (NF-κβ) and decreases the production of inflammatory cytokines. Its antioxidant property is attributed to its ability to increase antioxidant enzymes like catalase (CAT), superoxide dismutase (SOD), and glutathione (GSH), which are known to counteract oxidative damage. However, it is important to note that different parts of the plant had varying impacts on CVD risk factors, depending on whether the study was conducted preclinically or clinically. Therefore, its extract should be explored as a potential remedy for the management of CVD risk factors, especially in areas where access to healthcare is limited.

## 1. Introduction

Cardiometabolic diseases are a cluster of conditions that affect an individual’s cardiovascular system and metabolic health [1]. The relationship between cardiometabolic conditions and inflammation, as well as their impact on the progression of cardiovascular disease (CVD), is well established [2]. According to a Chinese data survey, the prevalence of these conditions was reportedly high in 2018, with diabetes mellitus (DM) at 11.9%, high blood pressure at 27.5%, and dyslipidemia at 35.6% [3]. This is supported by the International Diabetes Federation, which reported that 588.7 million individuals were living with DM in 2024; this number is anticipated to increase by 45% in 2025 [4]. This is more pronounced in low- and middle-income countries (LMICs). For instance, DM is anticipated to increase from 24.6 million in 2024 to 59.5 million in 2025 [4]. Similar observations have been noted with high blood pressure, where the prevalence increased from 11% in 2015 to 31.1% in 2024 [5]. Given that LMICs have minimal resources, the rapidly increasing prevalence of these conditions warrants an investigation of alternative remedies to manage them. The prevalence of these conditions could be worse, considering the large number of undiagnosed cases of DM and high blood pressure, especially in under-resourced communities [6,7].

Over the years, there have been improvements in modern medicine towards the management of CVDs and associated risk factors [8,9]. However, the global cost of controlling CVD is estimated to reach USD 1.4 billion by 2030 [10]. Due to these cost reasons, many people, especially in LMICs and under-resourced communities, still rely on traditional medicinal plants for their daily health needs, with some using traditional and modern medicine concurrently [11,12,13]. This heavy reliance on medicinal plants may be due to personal beliefs, and it is motivated by their potential safety profiles and availability. Moreover, limited access to healthcare services and the challenges of the affordability of medical care in LMICs seem to be contributing factors to the increasing reliance on medicinal plants [11]. Indeed, the setbacks of modern medicine, alongside the issues of affordability and accessibility, motivate the need for effective approaches to mitigate these conditions and their associated complications. One such important approach gaining interest is the use of plant extracts, with the morula tree, *Sclerocarya birrea* (*S. birrea*), being an example.

The marula tree is native to sub-Saharan Africa and bears edible fruits (marula fruits), and it is used in ethno-medical and cultural practices [14]. Its bark, leaves, and fruits have been used to treat DM, inflammation, and hypertension [14]. It is reported to contain bioactive compounds such as flavonoids, tannins, coumarins, and polyphenols, including vitamins A, C, and E, which exhibit antioxidant, anti-inflammatory, and antidiabetic properties [15,16,17,18,19]. However, evidence from rodents demonstrates contradicting findings. For instance, while the hypoglycemic effect of *S. birrea* was reported in diabetic rats based on fasting blood glucose (FBG), no effect was observed on glycated hemoglobin (HbA1c) or insulin [20], suggesting its potential limitation in regulating glucose metabolism. Other studies showed reduced FBG without any assessment of other markers of hyperglycemia following *S. birrea* administration in diabetic animal models [21,22].

Although *S. birrea* has shown promising preclinical evidence when using animal models of obesity and DM [23,24], there is limited translated clinical evidence demonstrating its efficacy in patients living with cardiometabolic diseases. Furthermore, the clear mechanism by which *S. birrea* offers cardioprotective effects is not fully documented. Therefore, this review aimed to document the cardioprotective potential of *S. birrea*, focusing on its effects on hyperglycemia, inflammation, dyslipidemia, hypertension, and oxidative stress.

## 2. Methodology

This is a narrative review study using evidence published in PubMed, Web of Science, Scopus, and ResearchGate. A comprehensive search for eligible literature was conducted for studies published from inception to 30 April 2025. The Boolean operators (AND) and (OR) were used to build the search. The following keywords were used to search for literature in the databases: “*Sclerocarya birrea*” OR “*S. birrea*” OR “Morula” AND “cardiometabolic disease” OR “diabetes mellitus” OR “hypertension” OR “dyslipidemia” OR “inflammation”. A manual search of the bibliography was conducted to identify relevant literature in published studies. Only peer-reviewed studies were included; gray literature (theses, dissertations, conference abstracts, letters, and review papers) were not considered. Clinical trials and evidence from preclinical studies (in vivo and in vitro) were included. Only studies that used *S. birrea* in any form of therapy and reported its effect on diabetes, inflammation, lipid profiles, blood pressure, and oxidative stress were reviewed. The present narrative review included preclinical and clinical studies that investigated the potential impact of *S. birrea* on cardiovascular health and related risk factors.

## 3. Results

### 3.1. Effect of S. birrea in In Vitro Studies

In vitro studies are important in research communities, as they help to assess the efficacy and safety profile of drugs, explore mechanisms, and create a background to allow for in vivo and clinical studies [25]. Five studies using cell lines, published between 2010 and 2020, were included (Table 1). These studies were conducted in three countries: three in South Africa [26,27,28], one in Cameroon [29], and one in Italy [16]. These studies used different parts of *S. birrea*, including the leaves, bark, or stem bark. In 3T3-L1 adipocyte cell lines and Cang liver cells, *S. birrea* treatment led to increased glucose uptake, concomitant with the inhibition of carbohydrases such as α-glucosidase [27]. Additionally, the same study showed an increased phenolic content in the stem bark of this extract. Similar findings were reported when C2C12, HepG2, and 3T3-L1 were treated with the methanolic extract of *S. birrea* [28]. Regarding the scavenging capacity, the methanolic extract of *S. berrea* showed a high potency [16,28]. The stem bark extract showed no effect on the expression of glucokinase, glucose transporter 2 (GLUT2), pyruvate carboxylase, or cyclooxygenase 1 (COX1) [29]. Notably, the cytotoxic activity of *S. birrea* was high in bark compared to in the leaf extract [16].

### 3.2. In Vivo Evidence Exploring the Effect of S. birrea in Obese and Diabetic Models

In vivo studies are important in research, as they validate in vitro findings and are further used as a baseline to investigate whether the evidence can be translated into the human body [30,31]. Twelve rodent studies [20,21,22,23,24,29,32,33,34,35,36,37] published between 2003 and 2025 were included in this review (Table 2). Five of the included studies were from South Africa [21,22,32,33,35]; four were from Cameroon [23,24,29,34]; and one was from Benin [37], Burkina Faso [36], and Nigeria [20]. Different rodents, including Wistar and Sprague Dawley rats and NMRI, C57BL/6J, and BALB/c mice, were used among the included preclinical studies. The human conditions in these rodents were mimicked by using diet (high-energy, -sucrose, and -fat diets and oxidized palm oil) or drugs such as streptozotocin (STZ), alloxan monohydrate, and nicotinamide.

### 3.3. Antidiabetic Potential of S. birrea Supplementation in Rodents

Hyperglycemia is one of the central features of DM, and it predisposes those living with DM to secondary complications, including endothelial dysfunction, kidney failure, retinopathy, and CVD [38,39]. The studies included in this review reported a positive effect of *S. birrea* supplementation on glucose metabolism. Notably, *S. birrea* significantly reduced fasting and postprandial glucose levels [20,21,22,23,24,29,33,34,35,36,37]. However, these findings were dose-dependent, with high doses of *S. birrea* having pronounced effects on glucose metabolism. For instance, the most effective *S. birrea* supplement in reducing FBG in diabetes rats was observed when the *S. birrea* stem bark was administered orally at a dose of 300 mg/kg for a period of 14 and 21 days [22,29,34]. Although its leaves and fruit peels showed promising results, they seemed to be dose-dependent, as higher doses (600 mg/kg) or longer durations (28 to 29 days) were required for effective results [21,32]. In addition, rodents supplemented with *S. birrea* experienced some significant improvements in glucose uptake and tolerance, as well as an increase in glucose-induced ATP generation and glucose oxidation [24,29,32,34]. For instance, Mabasa et al. showed that, in diabetic and obese rodents, 600 mg/kg of *S. birrea* leaves decreased blood glucose levels and improved glucose uptake [32]. Another report conducted in diabetic Wistar rats found that, when *S. birrea* bark extract was administered orally at a dose of 300 mg/kg for 21 days, there was a reduction in glycemia, improved glucose tolerance, and improved insulin sensitivity [24]. Consistently, *S. birrea* at doses of 300 and 600 mg/kg inhibited α-amylase and α-glucosidase and upregulated protein kinase β (PKβ) mRNA expression, thus preventing postprandial blood glucose rise and promoting intracellular glucose transport [22,32]. Insulin resistance has been reported to be a cardiovascular risk factor [40]. The evidence from this review showed that *S. birrea* supplementation can enhance insulin sensitivity, restore plasma insulin levels to near-normal values, and increase the insulin sensitivity index [24]. Interestingly, high doses were associated with a prominent impact. In addition, *S. birrea* supplementation was found to have a protective effect on β-cells against damage and stimulated insulin secretion [41,42]. DM-associated symptoms, such as polyphagia and polydipsia, were significantly reduced after S. *birrea* supplementation in diabetic rats [34]. Furthermore, *S. birrea* supplementation increased hepatic glycogen storage, indicating better energy utilization [23]. Specifically, a significant increase in hepatic glycogen was observed in diabetic rats treated with *S. birrea* at 150 mg/kg (86.30 ± 22.20 mg/g, *p* < 0.01) and 300 mg/kg (94.50 ± 12.30 mg/g, *p* < 0.001) compared to untreated diabetic rats (40.20 ± 5.06 mg/g) [23]. However, no effect was noted at a lower dose (75 mg/kg—44.10 ± 3.24 mg/g). Despite various doses (100, 200, and 400 mg/kg) of *S. birrea* having significant effects on blood glucose in diabetic rats, there was no effect on HbA1c, insulin, or glucose transporter-4 (GLUT4) [20]. Another experiment using 5 and 25 mg/kg of *S. birrea* bark revealed antihyperglycemic activities, as shown by reductions in blood glucose from 279.60 ± 36.25 to 86.00 ± 8.67 mg/dL in the diabetic rats in the 5 mg/kg group and from 279.60 ± 36.25 mg/dL to 86.33 ± 10.98 mg/dL in those in the 25 mg/kg group compared to the diabetic group without treatment (*p* < 0.001) [36].

### 3.4. Hypolipidemic Potential of S. birrea Supplementation in Rodents

Overall, *S. birrea* supplementation had a hypolipidemic effect in DM rodent models. This was observed in rodents that were treated with different doses of *S. birrea*, showing a significant decrease in TC, TG, LDL, and the atherogenic index (AIP) and an increase in HDL, thus reducing cardiovascular risk [21,24,32,34,37]. For instance, in obese Wistar rats, supplementation with *S. birrea* fruit peel (100–200 mg/kg) over 28 days led to a significant decrease in TC and TG and an increase in LDL levels [21]. Similar findings were reported in another experimental study, in which 150 to 300 mg/kg of *S. birrea* stem bark was used in diabetic rats [24]. The oral administration of *S. birrea* stem bark extract at doses of 150–300 mg/kg for 14 to 21 days led to a significant decrease in plasma TC, TG, LDL, and AIP levels and an increase in HDL in STZ and nicotinamide/STZ-induced diabetic Wistar rats [24,29,34]. Dimo et al. (2006) found that a higher dose (300 mg/kg) of *S. birrea* restored plasma TC and TG levels to near normal while also promoting weight gain, suggesting an improved metabolic status [34]. Similarly, a decrease in hepatic lipid accumulation was observed in obese and diabetic BKS.Cg-Dock7(m) +/+Lepr(db)/J mice after supplementation with 600 mg/kg of *S. birrea* leaf extract for 29 days [32]. This indicated an improvement in lipid metabolism and storage [32]. Additionally, 600 mg/kg of *S. birrea* supplementation downregulated fatty acid synthase (FAS) and upregulated peroxisome proliferator-activated receptor alpha (PPAR-α) and carnitine palmitoyl transferase 1 (CPT1), thus reducing lipogenesis and enhancing fatty acid β-oxidation in obese mice [32]. Another study in diabetic rats showed no effect of the hydroethanolic extract of *S. birrea* at different doses (100, 200, and 400 mg/kg) on lipid profiles, including TC, TG, LDL, and HDL [20].

### 3.5. Antioxidative and Anti-Inflammatory Potential of S. birrea Supplementation in Rodents

*S. birrea* contains high levels of epigallocatechin gallate and myricetin, flavonoids, and phenolic compounds, which possess antioxidative properties that promote antioxidant defense mechanisms [21,23,24,32]. The included studies demonstrated that *S. birrea* supplementation in DM and/or obese rodents exhibits antioxidative potential in a dose-dependent manner, as shown by a decrease in markers of oxidative stress, including lipid peroxidation, nitrite, and malondialdehyde (MDA), and the upregulation of antioxidant enzymes [23,24,32]. These enzymes, including catalase (CAT), superoxide dismutase (SOD), and glutathione (GSH), are known to counteract oxidative damage. Sewani-Rusike et al. (2021) [21] reported an increased total antioxidant capacity (TAC) following 28 days of *S. birrea* peel treatment in diabetic rodents. Consistently, MDA was also reduced concomitantly with increases in SOD, GSH, and CAT activities following *S. birrea* treatment [23,24]. Furthermore, Mabasa et al. (2022) reported the effect of 600 mg/kg of *S. birrea* at the genetic level in diabetic rodents and associated enzymes [32]. Firstly, *S. birrea* supplementation upregulates the expression of PPAR-α, which triggers the upregulation of CPT1. This promotes fatty acid oxidation, resulting in a decrease in hepatic fat and inflammation [32]. It is also associated with the suppression of FAS, which leads to the downregulation of the mRNA and protein level of sterol regulatory element-binding transcription factor 1 (SREBF1) [32,37]. This further inhibits de novo lipogenesis, resulting in a decrease in lipid accumulation, thereby reducing inflammation. Finally, *S. birrea* supplementation upregulates protein kinase-β (AKT). AKT in its upregulated form promotes insulin signaling and glucose uptake, leading to improvements in metabolic function and a decrease in inflammation [32]. *S. birrea* supplementation ameliorates inflammation through its lipid-lowering potential. In addition, *S. birrea* supplementation in diabetic rodents has been shown to reduce tumor necrosis factor-alpha (TNF-α) at all doses (75, 150, and 300 mg/kg), interferon gamma (INF-γ) only at 150 mg/kg, and interleukin-1 beta (IL-1β) at 150 and 300 mg/kg [23].

### 3.6. Antihypertensive Potential of S. birrea Supplementation in Rodents

Only three of the included studies reported the antihypertensive potential of *S. birrea* extract. All these studies support the antihypertensive potential of *S. birrea* in obese and diabetic models [21,24,33]. Notably, in diabetic rats, 150 and 300 mg/kg of *S. birrea* stem bark extract administration reduced MBP, SBP, and DBP [24]. This is supported by another experimental study in an animal model of diabetes, which showed that 60, 120, and 240 mg/kg of *S. birrea* reduces BP [33].

### 3.7. Overall Evidence from Clinical Studies

Only three clinical studies [43,44,45] published between 2008 and 2025 were found to be relevant and included in this review (Table 3). These studies were conducted in Burkina Faso, Spain, and Israel. One trial used an encapsulated *S. birrea* stem bark dry powder aqueous extract for 90 days among individuals living with prediabetes [43]. The other two trials used 200 mL (1 glass) of *S. birrea* (marula) juice and 180 mg in healthy individuals [44,45].

### 3.8. Antidiabetic Potential of S. birrea Supplementation in Humans

The administration of *S. birrea* had no significant effect on glycemia in healthy individuals (5.06 ± 0.30 compared to 5.28 ± 0.61, *p* = 0.18) [45]. Likewise, 200 mL of *S. birrea* did not change the blood glucose levels of healthy individuals (89 ± 8 compared to 88 ± 10, *p* > 0.05) [44]. The administration of *S. birrea* bark supplementation over 90 days in patients living with prediabetes caused no significant changes in HbA1c (5.24 ± 0.43 compared to 5.33 ± 0.44, *p* = 0.543) or FBG (102.9 ± 5.3 compared to 104.2 ± 5.0, *p* = 0.329) [43]. However, it caused significant improvements in insulin levels, HOMA-IR, and the quantitative insulin sensitivity check index (QUICKI index) and a decreased area under the curve of the oral glucose tolerance test (AUC of OGTT) [43]. Although bark supplementation did not seem to have a significant effect on FBG levels, *S. birrea* fruit juice supplementation had a significant impact on regulating glucose metabolism [43].

### 3.9. Hypolipidemic Potential of S. birrea Supplementation in Humans

The administration of *S. birrea* bark supplementation over 90 days in healthy individuals showed no statistically significant changes in serum TC, LDL, HDL, or TG [43]. In another trial in healthy individuals, *S. birrea* fruit juice supplementation had a significant impact on lipid profiles; however, some of these changes were not persistent in the post-intervention period [44]. For instance, this juice significantly reduced the concentrations of TC (*p* < 0.02) and LDL (*p* < 0.01) and increased HDL (*p* < 0.03). However, no effect was noted on TG during the 3 weeks of administration of marula juice. A recent finding by Coulidiaty et al. (2025) in healthy individuals demonstrated a significant 9% (0.91-fold) decrease in TC after *S. birrea* intervention (4.76 ± 0.88 to 4.33 ± 0.47, *p* < 0.01) [45]. In prediabetes, the ingestion of *S. birrea* powder showed no effect on lipid parameters, including TC, TG, LDL, and HDL (*p* > 0.05) [43].

### 3.10. Anti-Inflammatory Potential of S. birrea Supplementation in Humans

One of the markers of endothelial inflammation reviewed was E-selectin. Interestingly, the stem bark of *S. birrea* significantly reduced E-selectin levels, specifically resulting in a 1.12-fold decrease (baseline: 17.59 ± 1.11 ng/m; post-treatment: 15.70 ± 1.06 ng/m; *p* < 0.001) [43]. In addition, this review also focused on proinflammatory markers, including interleukin-6 (IL-6). However, there was no significant effect of *S. birrea* on IL-6 (*p* = 0.159) [43]. Furthermore, *S. birrea* supplementation inhibited LDL oxidation, which may prevent atherosclerosis [44].

### 3.11. The Antioxidative and Antihypertensive Potency of S. birrea in Humans

Different factors and markers were considered when determining the antioxidant capacity of *S. birrea*. These included the active phytochemical compounds and the scavenging activity of the extract. Notably, the evidence showed that *S. birrea* was rich in phenols and vitamin C, a widely active antioxidant agent. In addition, it showed strong free radical scavenging properties [44]. Marula juice also showed a high ferric reducing capacity (22 ± 7 mM) [44]. Contradictory findings were noted on blood pressure, especially in healthy individuals. One trial showed an antihypertensive effect [43], while another one showed no effect on either SBP or DBP [44]. In prediabetes, only SBP was reduced without an effect on DBP [43]. In addition, flow-mediated dilation (FMD) also increased, 8.61 ± 5.22% compared to 6.51 ± 5.11% (*p* < 0.040), suggesting a hypotensive effect.

### 3.12. Cytotoxicity and Safety Profile of S. birrea

Three studies were conducted to test for cell cytotoxicity and the sensitivity of *S. birrea* [16,29,33]. When administered to INS1E cells, the extract showed no acute side effects at the effective concentration; thus, *S. birrea* was proven to be safe in terms of cell integrity and differentiation, with no cytotoxicity observed at effective concentrations [29]. A lack of cytotoxicity and sensitivity to *S. birrea* treatment at higher concentrations was also observed in LLC-PK1 and MDBK cells [33]. However, another in vitro study, which used HepG2 and Human Dermal Fibroblasts, adult (HDFa) cells for a cytotoxicity analysis [16], reported higher cytotoxic activity of *S. birrea*, as demonstrated by pronounced alterations in cell morphology [16]. Furthermore, *S. birrea* induced cell death, associated with ROS generation, which led to a loss of mitochondrial membrane integrity, with subsequent cytochrome c release from the mitochondria into the cytosol [16]. In BALB/c mice, the lethal dose of 50% (LD50) values of aqueous and methanolic bark extracts of *S. birrea* were 1215 ± 38 mg/kg and 1087 ± 41 mg/kg, respectively, indicating that they were safe in this animal model [41]. Another preclinical study in healthy rats showed that *S. birrea* ethanolic extract, even at a higher dose (5000 mg/kg), was still safe, as no adverse events or behavioral changes were reported. Altogether, these findings suggest that *S. birrea* is a relatively safe remedy [46].

## 4. Discussion

**Figure 1:**
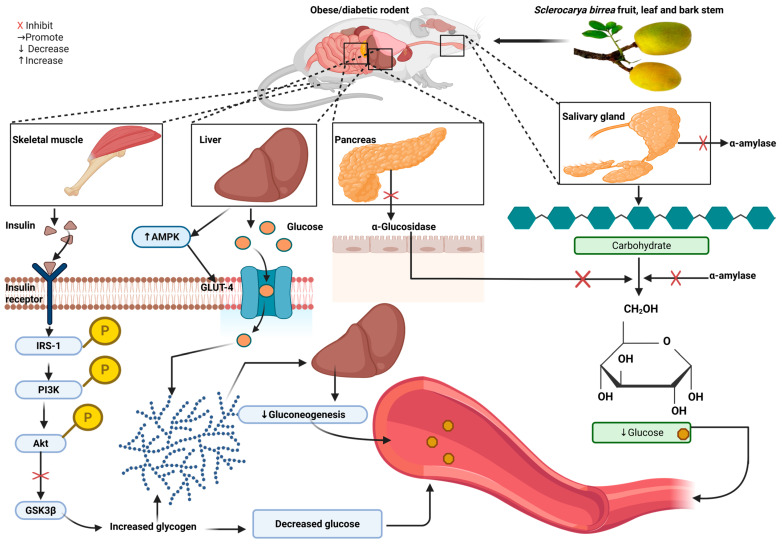
Proposed mechanism by which *S. birrea* ameliorates hyperglycemia [32]. *S. birrea* reduces hyperglycemia by inhibiting alpha-amylase and glucosidases. This action prevents the breakdown of carbohydrates into simple sugar molecules, resulting in reduced blood glucose levels. It also stores glucose as glycogen in the liver and skeletal muscle. Akt: protein kinase beta; IRS-1: insulin resistant substrate-1; GSK3-β: glycogen synthase kinase 3 beta; PI3K: phosphatidylinositol 3-kinase; AMPK: AMP-activated protein kinase; GLUT-4: glucose transporter type 4; PPAR: peroxisome proliferator-activated receptor. Figure was created using Biorender.

This study reviewed preclinical and clinical evidence assessing *S. birrea* as a remedy to control hyperglycemia, hyperlipidemia, inflammation, hypertension, and oxidative stress. A clear understanding of the potential effects of natural fruits and their active compounds can facilitate their use as alternatives in the management of CVD, especially in LMICs, where access to modern medicine is limited and the availability of these plants or fruits is abundant. The evidence in this review shows contradictory results between preclinical and clinical studies. In preclinical studies, *S. birrea* supplementation reduced hyperglycemia by reducing FBG and improving glucose uptake and tolerance [20,21,22,23,24,29,33,34,35,36,37]. It also improved insulin sensitivity and insulin deficiency-related symptoms, thus suggesting that *S. birrea* has a protective impact on some β-cells [23,24,29,34], with high doses showing a pronounced effect on glucose metabolism. Additionally, it showed inhibitory effects on carbohydrases (α-amylase and glucosidases) in diabetic models. Furthermore, it increased hepatic glycogen. While no effect was observed on HbA1c, insulin, or GLUT4 [20], this could be attributed to the *S. birrea* part used (leaves versus fruit peels), dosage, duration of supplementation, and method of extraction. Furthermore, most preclinical studies included in this review did not measure HbA1c, thus resulting in a lack of comparative evidence. In clinical studies, *S. birrea* fruit juice supplementation in healthy and prediabetic individuals had no significant effect in regulating serum glucose or HbA1c levels but did in improving FI, HOMA-IR, the QUICKI index, and 2 h post-OGTT [43,44,45]. This suggests that there is a potential limitation in the use of *S. birrea* as an antidiabetic agent in humans, as the preclinical findings on its antihyperglycemic effect are not reproduced in humans. Although there are conflicting findings, this might be due to limited evidence in humans, especially those living with diabetes, as the evidence in preclinical studies was collected from obese and diabetic animals. As an antihyperglycemic effect was noted in rodent models, it seems that *S. birrea* improves glucose metabolism through various mechanisms, including the inhibition of hepatic gluconeogenesis and the activation of adenosine monophosphate (AMP)-activated protein kinase, which enhances glucose uptake, reduces hepatic glucose production, and improves insulin sensitivity [21,23,37]. AMPK activation leads to the suppression of key gluconeogenic enzymes, such as phosphoenolpyruvate carboxykinase (PEPCK) and glucose-6-phosphatase [47]. This reduces the liver’s production of glucose from non-carbohydrate sources, thus reducing blood glucose. However, the inhibitory effect of *S. birrea* on α-amylase and α-glucosidases prevents the breakdown of carbohydrates into simple absorbable sugar molecules, hence reducing glucose in the bloodstream, further reducing hyperglycemia [22,27,28] (Figure 1).

**Figure 2:**
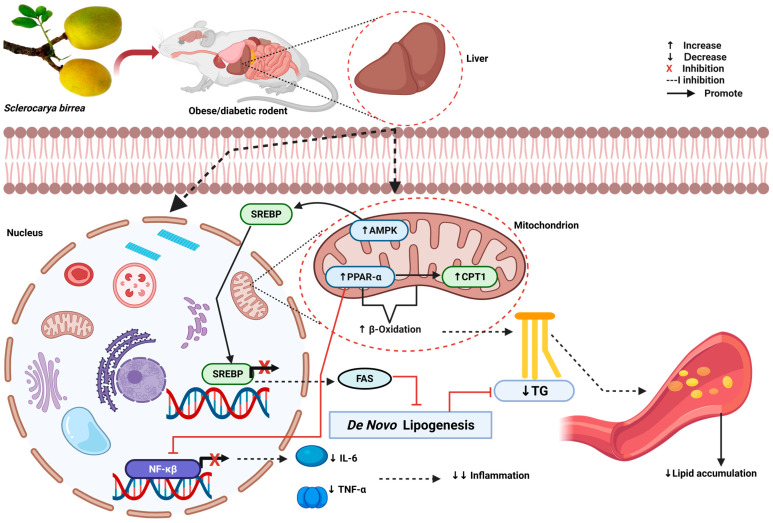
The possible mechanism by which *S. birrea* lowers inflammation and dyslipidemia [32,37]. SREBP: sterol regulatory element-binding protein 1, AMPK: AMP-activated protein kinase, CPT1: carnitine palmitoyl transferase 1, TG: triglyceride, FAS: fatty acid synthase, IL-6: interleukin-6, TNF-α: tumor necrosis factor alpha, NF-κβ: nuclear factor kappa beta. Figure was created via Biorender.

Evidence from preclinical studies showed that *S. birrea* supplementation had hypolipidemic properties, as demonstrated by a decrease in TC, TG, and LDL, alongside an increase in HDL coupled with a decrease in the AIP, reduced lipogenesis, and enhanced fatty acid β-oxidation [21,24,32,34,42]. These findings are supported by evidence from healthy individuals [43,44,45], although they are in contrast with that from prediabetes patients [43]. A recent in vivo study [20] also reported no hypolipidemic effect of the hydroethanolic extract of *S. birrea* leaves when compared to others. For instance, studies that reported a positive impact of *S. birrea* had a longer intervention duration and used different components of *S. birrea*, such as the bark and fruit peels [21,24,32,34,37]. This indicates that the part of *S. birrea* used and the period of intervention contribute to its lipid-lowering potential. Although the exact pathway that *S. birrea* targets in reducing lipid accumulation is not well documented, the effect appears to be mediated by its content of active compounds and nutrients, such as polyphenols, tannins, coumarins, flavonoids, triterpenoids, and phytosterols, due to their lipid-lowering potential [15,18,41]. However, other researchers suggest that *S. birrea* suppresses the enzymes FAS and SREBP-1 and further upregulates CPT1 levels and the expression of PPARα [32]. The activation of PPARα increases the expression of SREBP-1, thus promoting the synthesis of lipogenic genes such as FAS [48]. It is worth noting that SREBP-1 is a transcription factor that binds to FAS through the promoter region of the lipogenic gene, thus promoting the transcription of FAS genes and resulting in FA synthesis. Altogether, these activities reduce lipogenesis and enhance fatty acid β-oxidation [32]. Therefore, a decrease in SREBP-1 following *S. birrea* treatment suggests that FA synthesis is inhibited, resulting in reduced lipid accumulation [32,37] (Figure 2). Although in clinical studies, *S. birrea* showed conflicting findings in prediabetic and healthy individuals, we assume that this is attributed to the differences in the *S. birrea* part used; for instance, those that showed hypolipidemic effects were conducted using the leaves of the plant [44,45], while in prediabetic individuals, the use of the bark of *S. birrea* had no effect [43]. Key factors that might have contributed to these inconsistent results include the bioavailability and phytochemical components of *S. birrea* (bark compared to leaves or fruit juice), preparation methods of the supplement, dose and duration of intervention, and condition of the participants.

Evidence from preclinical studies indicates that *S. birrea* supplementation enhances antioxidant mechanisms by increasing free radical scavenging activity and ferric reducing antioxidant power and by upregulating antioxidant enzymes such as CAT, SOD, and GSH [16,23,24,44]. An increase in these enzymes, especially SOD, catalyzes the conversion of superoxide radicals into hydrogen peroxide and oxygen, hence protecting cells and tissues against free radicals [49]. However, GSH, an intracellular antioxidant, scavenges ROS and acts as a substrate for glutathione peroxidase (GPx), which reduces hydrogen peroxide to water [50]. MDA, a biomarker, is generated when ROS lipid peroxidation occurs [51]. An elevated level of MDA reduces GSH and inhibits SOD and CAT, thus reducing the ability to neutralize ROS molecules [49,52]. Interestingly, *S. birrea* reduced the level of MDA, supporting its antioxidant properties. This was also supported by the ability of *S. birrea* to reduce lipid peroxides [24,44]. The antioxidant properties of *S. birrea* are mediated by its high content of polyphenols, EGCG, myricetin, flavonoids, and vitamin C, as previously reported [16,21,32,44]. These phytochemicals reduce oxidative stress by improving the antioxidant defense mechanism through various pathways. For example, phenols activate the nuclear factor ethythroid-2-related factor 2 (Nrf2) pathway, which then upregulates the expression of SOD, CAT, and GPx [53]. Altogether, these enzymes suppress the production of ROS and reduce oxidative stress. Another notable signaling pathway by which *S. birrea* reduces oxidative stress is through the inhibition of the NADPH oxidase (NOX4) pathway [54]. NOX4 generates ROS by donating electrons from NADPH to oxygen, thus forming superoxide and hydrogen peroxide, resulting in oxidative stress. An NOX4 inhibition rate between 66 and 84% has been reported previously [54]. This is partly modulated by its high flavonoid content, which inhibits the activity of NOX4 and the generation of ROS, hence reducing oxidative stress [55]. Vitamin C also promotes an antioxidative environment by donating hydrogen atoms to neutralize ROS, including singlet oxygen, superoxide, and hydroxyl radicals, thus converting them into less reactive molecules [56]. The antioxidant activity of *S. birrea* is adequately confirmed in this study by its effect on antioxidant enzymes, including the content of phytochemical compounds.

*S. birrea* supplementation has shown anti-inflammatory effects in preclinical and clinical studies [23,43]. Preclinical evidence showed an anti-inflammatory effect demonstrated by reduced TNF-α, INF-γ, and IL-1β, alongside the activation of PPARα [23,43]. Clinical studies showed that the bark significantly reduced E-selectin levels, a marker of endothelial inflammation in prediabetes [43]. Although a potential anti-inflammatory effect was noted, evidence from preclinical and clinical studies focused on inflammation based on different markers. This makes it difficult to understand the mechanism behind the effect of *S. birrea* on inflammation. Nevertheless, according to existing evidence, the anti-inflammatory effect of *S. birrea* seems to occur through the mediation of different pathways. For instance, the activation of PPARα inhibits the nuclear translocation and DNA binding of nuclear factor kappa-beta (NF-κβ), thus blocking the transcription of inflammatory genes [32,37,57]. These subsequently reduce the production of inflammatory cytokines that promote inflammation [23] (Figure 2). Moreover, *S. birrea* inhibits the enzyme cyclooxygenase, which is involved in the synthesis of proinflammatory prostaglandins from arachidonic acid [58]. By blocking this enzyme, *S. birrea* reduces the production of prostaglandin and inflammation.

Preclinical antihypertensive effects of *S. birrea* were also reported, with contradictory findings [21,24,33]. Although clinical studies showed inconsistencies, only one study found no effect on blood pressure [44], while others reported hypotensive potential [21,23]. FMD is one of the noninvasive measures of endothelial function, reflecting the ability of blood vessels to dilate in response to an increased blood flow, mediated by nitric oxide [59]. Healthy endothelial function is characterized by increased FMD, which promotes vasodilation through NO production. However, reduced FMD impairs NO bioavailability, resulting in increased hypertension [60]. Hence, improved FMD following *S. birrea* treatment in prediabetic patients supports its antihypertensive potential [43]. One of the essential signaling pathways by which *S. birrea* exhibits its hypotensive potential is through the activation of Akt, as this promotes NO production, resulting in vasorelaxation [32]. *S. birrea* is rich in potassium, which reduces blood pressure by promoting vasodilation and sodium excretion [44]. Due to its high content of flavonoids and tannins, *S. birrea* reduces oxidative stress and improves endothelial function [19]. These activities together contribute to vasodilation and lower blood pressure.

Some reported a dose-dependent relaxation of norepinephrine (NE)- and 5-hydroxytryptamine (5-HT)-induced contractions in the endothelium, along with an improvement in the mean arterial pressure [33]. While the clinical evidence in this study appears to contradict the findings of preclinical studies, it is essential to acknowledge that only three clinical studies with varying conditions were reviewed, which limits our clinical interpretation. Therefore, this evidence warrants future studies to explore *S. birrea* in clinical settings, validating the preclinical findings and confirming their translatability into clinical settings. It is worth noting that the safety profile of *S. birrea* was confirmed in cell cultures to gain a better understanding of its cytotoxicity.

### Limitations of the Studies

The selected studies used different induction methods. Some preclinical studies used mice, while others used rats; the process of inducing the conditions varied (drugs and diet). Various strains of rodents were used, ranging from Wistar rats to NMRI mice, Sprague Dawley rats, C57BL/6L and BALB/c mice. Different parts of *S. birrea* (leaves, fruit peels, and stem bark) were used, with varying methods of extraction or solvents (water, ethanol, hexane, and methanol), doses, and durations. There were limited clinical trials in this area, and those included had variations, with one conducted amongst prediabetic patients and the others conducted in healthy individuals. Furthermore, the authors acknowledge that translating the effect of *S. birrea* from preclinical models to clinical settings may present challenges. For instance, the bioavailability and physiological complexities of humans compared to animal models can make it difficult to translate preclinical results to clinical settings.

## 5. Conclusions and Future Directions

The evidence in this study suggests that *S. birrea* has the potential to improve cardiometabolic health. Evidence from preclinical studies revealed that *S. birrea* supplementation improved glucose metabolism and ameliorated hyperglycemia. In clinical studies, *S. birrea* improved fasting insulin, HOMA-IR, and the QUICKI index, and it decreased the AUC of OGTT; however, it had no notable effect on blood glucose. In addition, *S. birrea* supplementation exhibited antihyperlipidemic potential clinically and preclinically. *S. birrea* supplementation demonstrated an anti-inflammatory effect in both preclinical and clinical studies. The antioxidant properties of *S. birrea* were also noted through its strong free radical scavenging ability. The antihypertensive effect of *S. birrea* was also observed. However, as most evidence presented in this study was gathered from preclinical studies, we recommend that this natural supplement be explored in robust, large-scale, randomized controlled trials in humans to validate the preclinical findings reported.

## Figures and Tables

**Table 1:** Summary of evidence from preclinical (in vitro) studies.

Reference	Country	Experimental Model and Interventions	Experimental Findings
Kgopa et al., 2020 [26]	South Africa	Insulinoma cells, H-4-II-E liver cells. Cells treated with 50 µg/mL of *S. birrea* stem bark extract.	Dose-dependent increase in glucose uptake, enhanced insulin synthesis, and glucose-stimulated insulin secretion. Upregulation of glucose synthase.
Matsabisa et al., 2019 [27]	South Africa	3T3-L1 adipocytes and Chang liver cells. 50 μg/mL of stem bark *S. birrea* extract (methanol, hexane, and DCM).	Inhibited α-glucosidase and high phenolic content. Increased glucose uptake and AGEs.
Russo et al., 2018 [16]	Italy	HepG2 and HDFa cells were treated with bark and leaf methanol extracts of *S. birrea* (10, 50, 100, 200, and 300 μg/mL) for 24 h.	Higher ROS, polyphenol, tannin, and cytotoxic activities in *S. birrea* bark extract than in the leaf. Higher scavenging activity of methanol.
Mousinho et al., 2013 [28]	South Africa	C2C12, HepG2, and 3T3-L1 cells were treated with 1.56–6.25 μg/mL of bark of *S. birrea* extract (aqueous and methanol).	Methanol extract showed strong scavenging activity. Increased glucose uptake. Inhibited α-amylase and α-glucosidase
Ndifossap et al., 2010 [29]	Cameroon	INS-1E cells were treated with 5 μg/mL of *S. birrea* stem bark extract for 24 h.	Increased glucose oxidation and ATP generation. No effect on glucokinase, GLUT2, pyruvate carboxylase, or COX1.

HDFa: Human Dermal Fibroblasts, adult; HepG2: human hepatocellular carcinoma cell; ROS: reactive oxygen species; COX1: cyclooxygenase-1; GLUT2: glucose transporter 2; ATP: adenosine triphosphate; AGEs: advanced glycation end products; DCM: dichloromethane.

**Table 2:** Summary of evidence from preclinical (in vivo) studies.

Reference	Country	Experimental Model, Dose and Duration of Intervention	Hypoglycemic Activities	Hypolipidemic Activities	Anti-Inflammatory Antioxidative Stress Activities	Hypertensive Activities
Coulidiaty et al., 2025 [20]	Nigeria	High fructose-fed, streptozotocin (STZ)-induced type 2 diabetes (T2D) in Wistar rats. 100, 200, and 400 mg/kg of hydroethanolic extract of *S. birrea* leaves, administered orally for 21 days.	All doses reduced blood glucose. No effect on HbA1c, insulin, or GLUT-4.	No effect on TC, TG, LDL, or HDL	NS	NS
Traoré et al., 2024 [36]	Burkina Faso	Alloxan monohydrate-induced diabetes in Wistar rats. 5 and 25 mg/kg of *S. birrea* bark aqueous extract for 4 weeks.	Reduced FBG.	5 mg/kg increased TC, LDL, and HDL without effect on TG. 25 mg/kg decreased TG and LDL.	NS	NS
Tientcheu et al., 2023 [23]	Cameroon	Fructose-fed, STZ-induced diabetes in Wistar rats. 75, 150, and 300 mg/kg of stem barks of *S. birrea* and *Nauclea latifolia plus* fruits of *Piper longum* (SNP) for 21 days.	150 and 300 mg/kg decreased glucose. All doses decreased HOMA-IR. Increased insulin and hepatic glycogen.	NS	Decreased TNF-α, IL-1β, INF-γ with 150 mg/kg. MDA decreased. CAT increased. GSH increased at 150 and 300 mg/kg.	NS
Mabasa et al., 2022 [32]	South Africa	Nine-week-old diabetic BKS.Cg-Dock7(m) +/+ Lepr(db)/J obese mice on standard rodent maintenance diet. 600 mg/kg of *S. birrea* leaves, macerated in de-ionized water, was administered orally for 29 days.	Decreased non-FBG levels, upregulated Akt. No effect on PI3K, AMPK, or GLUT2.	Decreased hepatic lipid accumulation, FAS Upregulated expression of PPARα and CPT1, enhancing β-oxidation of fatty acids.	Contains high EGCG and myricetin content.	NS
Sewani-Rusike et al., 2021 [21]	South Africa	High-energy diet (HED)-induced obesity in Wistar rats. 100–200 mg/kg of *S. birrea* fruit peel hydro-ethanolic extract administered orally for 28 days.	Decreased FBG, insulin, and HOMA-IR. Enhanced glucose tolerance.	Decreased TC, TG, and LDL at both doses.	High polyphenol, flavonoid, and TAC content in fruit peel compared to pulp.	Significant decrease in BP at both doses compared to the untreated obese.
Ngueguim et al., 2016 [24]	Cameroon	Oxidized palm oil and sucrose induced hyperglycemia in albino Wistar rats. 150 and 300 mg/kg of *S. birrea* stem bark were administered orally for 21 days.	Both doses decreased blood glucose. Increased insulin sensitivity index.	Decreased TG, LDL, TC, and AIP. Increased HDL.	Decreased lipid peroxidation, nitrite levels, and MDA in the liver. Increased SOD and GSH in the liver and kidney.	Both doses reduced SBP, DBP, and MBP.
Attakpa et al., 2015 [37]	Benin	High-fat-diet-fed C57BL/6J mice. 200 or 300 mg/kg stem bark extract for 10 weeks.	Both doses reduced non-FBG. 300 mg/kg reduced insulin. Increased Akt, AMPK.	Decreased TG, FFA, and SREBP-1. Increased PPARα expression.	NS	NS
Mogale et al., 2011 [22]	South Africa	Alloxan monohydrate-induced DM in albino Wistar rats. 300 mg/kg of *S. birrea* bark aqueous, methanolic, acetone, and hexane extracts were administered orally.	Inhibited α-amylase and α-glucosidase. Reduced postprandial blood glucose levels.	NS	NS	NS
Ndifossap et al., 2010 [29]	Cameroon	Nicotinamide and STZ-induced DM in Wistar rats. 150 or 300 mg/kg of *S. birrea* stem bark extract was administered orally for 14 days.	Decreased FBG, restored insulin levels.	NS	NS	NS
Gondwe et al., 2008 [33]	South Africa	STZ-induced DM in Wistar rats. 60, 120, and 240 mg/kg of *S. birrea* bark ethanol extract administered orally for 5 weeks.	Reduced blood glucose and increased hepatic glycogen. No effect on insulin.	NS	NS	Reduced mean arterial pressure.
Dimo et al., 2006 [34]	Cameroon	STZ-induced DM in Wistar rats, with 150 and 300 mg/kg methanolic/methylene chloride extract of *S. birrea* stem bark administered orally for 21 days.	Decreased blood glucose levels, polyphagia, and polydipsia. Increased plasma insulin.	TC and TG levels returned to normal at a dose of 300 mg/kg.	NS	NS
Ojewole et al., 2003 [35]	South Africa	STZ-induced DM in Balb C mice and Wistar rats. 100–800 mg/kg methanol/methylene chloride extract of *S. birrea* stem bark administered orally for 8 h.	Decreased FBG levels.	NS	NS	NS

DM: diabetes mellitus; STZ: streptozotocin; FAS: fatty acid synthase; FFA: free fatty acid; EGCG: epigallocatechin-3-gallate; TC: total cholesterol; TG: triglyceride; HDL: high density lipoprotein; LDL: low density lipoprotein; CAT: catalase; SOD: superoxide dismutase; MDA: malondialdehyde; GSH: reduced glutathione; FBG: fasting blood glucose; HbA1c: glycated hemoglobin; HOMA-IR: homeostatic model assessment of insulin resistance; GLUT-4: glucose transporter 4; AIP: atherogenic index of plasma; PI3K: phosphatidyl-inositol-3-kinases; Akt: protein kinase beta; AMPK: AMP-activated protein kinase; GLUT2: glucose transporter type 2; PPAR: peroxisome proliferator-activated receptor; SREBP: sterol regulatory element-binding protein 1; CPT1: carnitine palmitoyl transferase 1; TNF-α: tumor necrosis factor alpha; NS: not stated; BP: blood pressure, MBP: men blood pressure; SBP: systolic blood pressure; DBP: diastolic blood pressure.

**Table 3:** Summary of evidence from clinical studies.

Reference and Country	Experimental Design	*S. birrea* Dose and Duration of Intervention	Antidiabetic Activities	Hypolipidemic Activities	Inflammatory and Antioxidant Activities	Antihypertensive Activities
Coulidiaty et al., 2025 [45] Burkina Faso	Phase I open-label clinical trial 10 healthy males, aged 18 to 40 years	Oral administration of 1800 mg of *S. birrea* leaf powder for 14 days	No significant changes in glycemia	Decreased TC	NS	Reduced BP
Victoria-Montesinos et al., 2021 [43] Spain	Randomized, double-blind, placebo-controlled trial 67 patients with prediabetes (33 on *S. birrea* and 34 on placebo), aged 18 to 65 years	Daily ingestion of 100 mg *S. birrea* stem bark aqueous powder per day for 90 days	No change in FBG or HbA1c Increased FI, HOMA-IR, and QUICKI index Decreased 2 h post-OGTT	No significant changes in serum TC, LDL, HDL, or TG	Decreased E-selectin No effect on IL-6 levels	Reduced SBP No change in DBP Improved FMD
Borochov-Neori et al., 2008 [44] Israel	Phase I open-label, non-controlled clinical trial 10 healthy males aged 18 to 40 years	200 mL of *S. birrea* juice per day for 3 weeks	No effect on serum glucose levels	Decreased TC, LDL, and TG, increased HDL	Inhibited ox-LDL Reduced lipid peroxides High in vitamin C, phenols, and increased FRAP Strong free radical scavenging ability	No effect on SBP or DBP

BP: blood pressure; DBP: diastolic blood pressure; FBG: fasting blood glucose; FI: fasting insulin; FMD: flow-mediated dilation; FRAP: ferric reducing antioxidant power; HbA1c: glycated hemoglobin; HDL: high-density lipoprotein; HOMA-IR: homeostatic model assessment of insulin resistance; LDL: low-density lipoprotein; NS: not stated; 2 h post-OGTT: 2-h post-oral glucose tolerance test; QUICKI: quantitative insulin sensitivity check index; SBP: systolic blood pressure; TC: total cholesterol; TG: triglyceride.

## Data Availability

All data generated in the present work are included in the manuscript.
